# FoodBD: a polygon-annotated meal image dataset of Bangladeshi cuisines with visual and nutritional labels

**DOI:** 10.1186/s13104-025-07583-8

**Published:** 2025-12-09

**Authors:** Benzir Md Ahmed, Md. Enamul Haque, A. K. Obidul Huq, Mohammad Mehedy Masud, Mohammed Eunus Ali, Mahmuda Naznin

**Affiliations:** 1 Department of Computer Science and Engineering, Bangladesh University of Engineering and Technology, Dhaka, 1000 Bangladesh; 2 College of IT, United Arab Emirates University, Al Ain, 15551 United Arab Emirates; 3 Monash University, Wellington Rd, Clayton, Melbourne, VIC 3800 Australia; 4 Department of Food Technology and Nutritional Science, Mawlana Bhashani Science and Technology University, Santosh, Tangail, 1902 Bangladesh; 5 Department of Computer Science and Engineering, United International University, Dhaka, 1212 Bangladesh

**Keywords:** Bangladeshi food, Food nutrition estimation, Computer vision, Image segmentation, Deep learning, Dietary assessment, Diabetes management

## Abstract

**Objectives:**

The FoodBD dataset was initially collected to address the dietary assessment of diabetic patients. However, it was later expanded to address the lack of culturally diverse food image datasets, particularly for Bangladeshi cuisine, which is underrepresented in food recognition research. It supports tasks in computer vision, nutrition estimation, and health monitoring by providing a resource for AI-driven dietary assessment tools.

**Data description:**

FoodBD comprises 3,523 smartphone-captured meal images representing authentic Bangladeshi meals, with minimal preprocessing to preserve real-world complexity. Each image is annotated with polygon-based segmentation across 67 food categories. Additionally, among them 1,837 images include expert-estimated nutritional information (carbohydrate, protein, fat, fiber, calorie, and glycemic load). The dataset is split into training, validation, and test subsets, facilitating reproducibility in machine learning pipelines.

## Objective

Accurate dietary assessment plays a critical role in health monitoring, chronic disease management, and nutritional research. In recent years, the use of image-based meal tracking and food recognition has gained traction because of the increasing availability of mobile cameras and advances in computer vision. However, the effectiveness of such AI-driven systems is highly dependent on the quality and diversity of the training data they use. Most publicly available food image datasets feature primarily Western [1, 2], East Asian [3–5], or mixed cultural [6–8] cuisines. This limits their applicability in regions such as Bangladesh, where food preparation, ingredients, and presentation differ significantly from those of the above mentioned cuisines. The FoodBD dataset was developed to address this challenge.

The primary objective of the FoodBD dataset is to support dietary assessment for diabetic patients in Bangladesh. This goal was later broadened to develop a high-quality, culturally contextualized dataset of Bangladeshi meals, enabling applications in both computer vision and health research. This dataset aims to help develop multimodal AI models for nutrition estimation, dietary monitoring, and chronic disease management. It was not previously published as part of a research paper, as the focus was specifically on dataset collection, annotation, and availability for reuse.

## Data description

The dataset was collected from multiple locations across Dhaka, Bangladesh. The dataset contains a total of 3,523 smartphone-captured meal images collected under real-world conditions, covering breakfast, lunch, and dinner. These images were captured by participants using their mobile devices, resulting in natural variability in resolution, lighting, and angles. To ensure usability, the images were standardized by resizing to 1024 × 1024 pixels using Roboflow’s [9] “Fit (black edges)” option, which preserves the original aspect ratio without cropping or distortion.

Each meal image is accompanied by detailed polygon-based annotations representing food items. All annotations were performed manually using the LabelMe [10] tool, following a standardized annotation protocol to ensure consistency in polygon boundaries and labeling style. To verify the quality of the annotations, multiple validation steps were implemented. First, faculty members from the Department of Food Technology and Nutritional Science reviewed each batch of annotations and provided domain-specific feedback on food category distinctions and portion boundaries. Second, a random subset of approximately 30% of the annotated images was cross-checked by a separate annotator to confirm boundary precision and class correctness. Finally, discrepancies identified during this review were resolved through discussion and expert input, ensuring high inter-annotator agreement. This multi-step validation process strengthened the consistency and reliability of the polygon annotations across the dataset.

A total of 12,575 annotations span 67 food classes, including both common staples (such as rice, ruti, fish, chicken, and lentils) and less frequently consumed items (such as mango, guava, and sweets). Food labels were recorded in both Bangla and English, ensuring cultural relevance while remaining accessible to an international research audience. The annotation files are stored in plain-text format, with each line representing one food item through a class ID followed by polygon coordinates (x, y) (see Dataset 2). A representative example of this file is also provided as a PNG image (see Data file 4) in the metadata for illustration purposes. Data file 3 illustrates the folder structure, whereas Data file 5 depicts the data collection and annotation workflow. Examples of original and annotated images are presented in Data file 7, which provides a visual result of the annotation process.

In addition to visual annotations, 1,837 images were enriched with nutritional metadata. These annotations were provided by a nutrition expert who carefully reviewed each meal image and (i) estimated the portion sizes of visible ingredients on the basis of dietary guidelines and professional expertise; ii) assigned nutritional values per serving covering carbohydrate (CHO), protein, fat, fiber, and calorie using standardized data sources [11, 12]; iii) obtained the glycemic index (GI) of individual ingredients from [13]; iv) calculated the glycemic load (GL) following the formula GL = (GI × weight)/100, as outlined in [14], to provide a more accurate indicator, as it incorporates both the GI and the estimated weight (in grams) of each ingredient, and v) finally aggregated ingredient-level estimates to yield total CHO, protein, fat, fiber, and calorie values per image.

Standard serving guidelines specific to the Bangladeshi population were followed to ensure consistency in portion size estimation. These guidelines are based on indigenous household utensils and traditional food preparation practices, as first recommended by Ali et al. [15] and later endorsed in the book chapter “Serving Size and Food Exchange List” supported by the Ministry of Planning, Government of the People’s Republic of Bangladesh [12]. In addition to these standardized references, common household measures and visual cues (e.g., standardized plates, bowls, and spoons) were used during the annotation process to estimate portion sizes as accurately as possible. These details are intended to make the procedure transparent and reproducible for other researchers. The dual nature of FoodBD, as both an annotated image dataset and a nutritional dataset, supports a wide range of downstream applications, including calorie estimation, dietary monitoring, diabetes management, and AI-powered nutrition recommendation systems.

The dataset has been systematically organized into training (70%), validation (20%), and test (10%) splits to support standardized machine learning workflows. Each subset is placed in a dedicated directory with separate folders for images and annotations. A consistent naming convention, FoodBD-XXXX, ensures traceability between images and their annotation files. Metadata describing food instances, environmental conditions, and preprocessing details are compiled in Data file 1. The nutritional annotations are stored in Data file 2, which contains detailed macronutrient and GL values for each meal image.

A summary of all dataset components and figures is presented in Table 1. This table lists the images, annotation files, metadata, nutritional files, and supporting figures, enabling researchers to easily locate the relevant resources in the repository.

**Table 1:** Overview of data files/datasets

Label	Name of data file/dataset	File types (file extension)	Data repository and identifier (DOI or accession number)
Dataset 1	FoodBD meal images	.jpg	Mendeley (10.17632/xh3ghf3jbg.2) [16]
Dataset 2	Polygon-based annotations	.txt	Mendeley (10.17632/xh3ghf3jbg.2) [16]
Data file 1	FoodBD_Meta_data.csv	.csv	Mendeley (10.17632/xh3ghf3jbg.2) [16]
Data file 2	MealNutrition1837_All.csv	.csv	Mendeley (10.17632/xh3ghf3jbg.2) [16]
Data file 3	Dataset_Folder_Structure	.png	Mendeley (10.17632/xh3ghf3jbg.2) [16]
Data file 4	Single_Meal_Annotation_File	.png	Mendeley (10.17632/xh3ghf3jbg.2) [16]
Data file 5	Data_Collection_Pipeline	.png	Mendeley (10.17632/xh3ghf3jbg.2) [16]
Data file 6	Class_Distribution_Chart	.png	Mendeley (10.17632/xh3ghf3jbg.2) [16]
Data file 7	Original_vs_Annotated_Images	.png	Mendeley (10.17632/xh3ghf3jbg.2) [16]

## Limitations

- Class imbalance, with some foods (e.g., rice, ruti, and chicken) overrepresented whereas others (e.g., guava, mango, and roll) are underrepresented (see Data file 6).
- Variability in image quality due to the use of different mobile devices, lighting, and angles.
- Manual annotations may contain minor human errors or subjective interpretations of food boundaries.
- In this dataset, glycemic load (GL) was calculated using the total weight of the food item rather than the grams of available carbohydrate. While this provides an approximate GL estimate, it may differ from values derived from available carbohydrate content, which is the conventional method in nutritional research. This limitation should be considered when interpreting or using the GL values in further analyses.

To address class imbalance, future users may apply data augmentation techniques such as horizontal or vertical flipping, rotation, color jittering, and brightness normalization, as well as balancing strategies like oversampling or weighted loss functions. Additionally, users may filter images based on resolution or lighting conditions to mitigate quality variations. Incorporating such preprocessing steps can help ensure more robust model training and improve the overall performance of subsequent analyses.

## Data Availability

The data described in this Data note can be freely and openly accessed on [Mendeley] at 10.17632/xh3ghf3jbg.2. Please see Table 1 and reference [16] for details and links to the data.

## Abbreviations

CHO: Carbohydrate
GL: Glycemic Load
GI: Glycemic Index
